# Outcome of subtotal parathyroidectomy for surgical treatment of hyperparathyroidism after renal transplantation^☆^^☆☆^^☆^

**DOI:** 10.1016/j.bjorl.2025.101623

**Published:** 2025-06-04

**Authors:** Murilo Catafesta das Neves, Ana Beatriz Ribeiro Fonseca, Camila Akemi Yamashiro Koike, Davi Knoll Ribeiro, Mayra Messias Lera, Rodrigo Oliveira Santos

**Affiliations:** Universidade Federal de São Paulo (UNIFESP), Departamento de Otorrinolaringologia e Cirurgia de Cabeça e Pescoço, São Paulo, SP, Brazil

**Keywords:** Hyperparathyroidism, Parathyroidectomy, Renal transplantation, Hypercalcemia

## Abstract

**Objective:**

To evaluate the efficacy of STPX as a definitive treatment for Hyperparathyroidism after Renal Transplantation (HPT-RT) and determine whether there is a relationship between percentage intraoperative Parathyroid Hormone (ioPTH) decay levels and Parathyroid Hormone (PTH) values on the first postoperative day and surgical success.

**Methods:**

This retrospective study analyzed the medical records of prospectively followed patients diagnosed with HPT-RT submitted to STPX in two tertiary hospitals (Brazilian Unified Health System ‒ SUS) for two years. Patients were allocated in two groups according to operative success (SG ‒ normalization of ionized Calcium [iCa] and PTH six months after surgery) or operative failure (FG ‒ persistence of high iCa and PTH). The percentage ioPTH decay levels and the postoperative PTH absolute values on the first postoperative day were evaluated in both groups.

**Results:**

Of the total sample of 31 patients, surgical treatment was successful in 27 cases (87%). No statistically significant differences were observed between the groups for the percentage ioPTH decay levels (SG = 71.51% vs. FG = 70.4%), as well as for the PTH absolute values on the first postoperative day (SG = 52.69 pg/mL vs. FG = 54.55 pg/mL).

**Conclusion:**

Subtotal parathyroidectomy is an effective and safe procedure for treating HPT-RT; however, the use of PTH levels remains subjective and cannot be considered as a predictor of surgical success.

## Highlights

• No statistically significant differences were observed between success and failure groups for the percentage intraoperative decay levels, as well as for the PTH absolute values on the first postoperative day.
• Subtotal parathyroidectomy is an effective and safe procedure for treating renal transplant parathyroidectomy.
• PTH levels remain subjective and cannot be considered as a predictor of surgical success.

## Introduction

The term Tertiary Hyperparathyroidism (THPT) refers to persistent Hyperparathyroidism (HPT) after successful Renal Transplantation (RT),^1^, ^2^ and it is observed in approximately 17%‒50% of patients with RT.^1^, ^3^, ^4^, ^5^ Hyperparathyroidism after Renal Transplantation (HPT-RT) is a morbid condition that can lead to various serious complications such as osteoporosis, pathological bone fractures, nephrolithiasis and nephrocalcinosis, which may cause renal dysfunction and graft loss.^3^

Drug treatment is possible and aims to control Parathyroid Hormone (PTH) and serum calcium levels. Its main therapeutic line is based on calcimimetic drugs, which act on the Calcium-Sensing Receptors (CaSR) of the parathyroid glands by suppressing PTH secretion. However, up to 50% of patients present Gastrointestinal Intolerance (GI) to these medicines and their efficiency is reduced in cases of most advanced HPT-RT.^5^, ^6^

Parathyroidectomy is the surgical treatment option.^7^ In addition to resolving HPT-RT, this surgical technique presents benefits to the heart and bones, as well as to the transplanted kidney, in addition to being a safe and effective therapy with no significant effects on long-term graft survival.^1^, ^4^, ^8^, ^9^ Although surgical indications are well determined,^10^ choice of the best surgical technique (total parathyroidectomy alone or with autograft or subtotal parathyroidectomy) is still controversial.^1^, ^11^

The use of intraoperative Parathyroid Hormone (ioPTH) is well established in cases of primary HPT^12^ and total parathyroidectomy due to the HPT secondary to chronic kidney disease.^13^ For these situations, the use of ioPTH is a good predictor of surgical success. However, few series address its use in cases of subtotal surgeries, as well as its ability to predict surgical success.

This study aimed to evaluate the efficacy of Subtotal Parathyroidectomy (STPX) as a definitive treatment for HPT-RT and determine whether ioPTH can be used as a predictive tool for surgical treatment success.

## Methods

After being approved by the institutional review board, a retrospective cohort study was prospectively conducted in two tertiary hospitals (Brazilian Unified Health System ‒ SUS): Euryclides de Jesus Zerbini Transplant Hospital and Kidney and Hypertension Hospital, from June 2016 to May 2018. Inclusion criteria comprised patients diagnosed with HPT-RT submitted to STPX with intraoperative localization of the four parathyroid glands, which were regularly followed postoperatively. Indications for surgical intervention were based on the criteria of the Brazilian Society of Nephrology (SBN).^10^

Surgical strategy for all patients was based on identification of the four parathyroid glands and preservation of parathyroid tissue corresponding to the volume of two normal glands (approximately 100 mm^3^).^14^ Peripheral ioPTH was collected at Time-0 (T0), during anesthetic induction, and at Time-20 (T20), 20 min after removing the last gland. Follow-up was performed with collection for laboratory tests on the first postoperative day and one, three and six months after surgery, according to the routine return visits determined by the nephrology team. The values of iCa and PTH were used in the analysis.

Patients were divided into two groups according to surgical success during the six-month follow-up. The Success Group (SG) included patients with resolution of hypercalcemia (iCa < 1.40 mmoL/L at the Euryclides de Jesus Zerbini Transplant Hospital and iCa < 1.35 mmoL/L at the Kidney and Hypertension Hospital) during follow-up. The Failure Group (FG) was composed of patients with persistent high iCa and PTH values (PTH > 68 pg/mL) in this period.

Statistical analysis was performed for both groups regarding percentage ioPTH decay and PTH absolute value on the first postoperative day in an attempt to correlate them with surgical success.

For statistical analysis, the numerical variables were expressed as mean and standard deviation or minimum and maximum values, whereas the categorical variables were expressed as number and percentage. The D’Agostino-Pearson normality test and the Shapiro-Wilk test were performed to verify the homoscedasticity (constant variance) between both groups. The Wilcoxon matched-pairs signed rank test and the Mann-Whitney test were used to compare the nonparametric variables, considering *p* < 0.05 as statistically significant value using SPSS 20.0 (SPSS Inc; Ilinois, USA) and using GraphPad Prism 4.1 software (CA 92037 USA).

## Results

The study included 31 patients (16 male and 15 female), with a mean age of 50.8 ± 8.1 years, divided into two groups according to surgical success, defined by PTH and iCa values (“success group” and “failure group”).

**Fig. 1:**
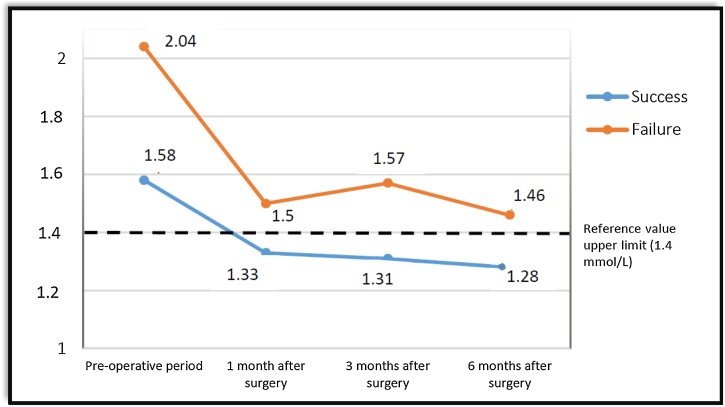
Variation of ionized Calcium (iCa) levels in patients operated at the Euryclides de Jesus Zerbini Transplant Hospital.

**Fig. 2:**
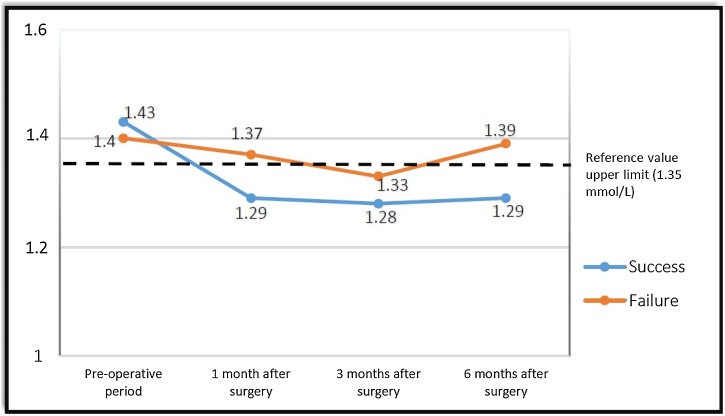
Variation of ionized Calcium (iCa) levels in patients operated at the Kidney and Hypertension Hospital.

**Fig. 3:**
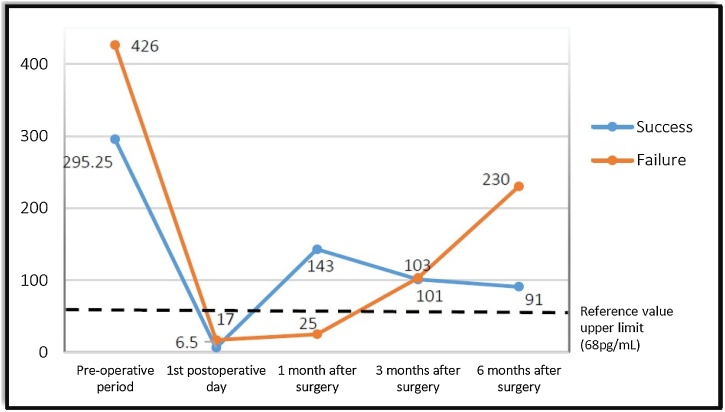
Variation of PTH values in patients operated at the Euryclides de Jesus Zerbini Transplant Hospital.

**Fig. 4:**
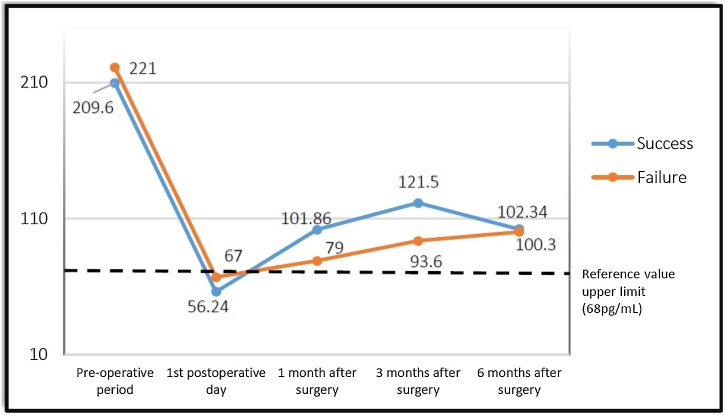
Variation of PTH values in patients operated at the Kidney and Hypertension Hospital.

After six months of postoperative follow-up, it could be noted that surgical success was observed in 27 patients (87%), however, surgical success was not observed in four patients (13%). There were no cases of hypoparathyroidism or loss of renal graft. The mean of pre and postoperative PTH and iCa values of the groups are shown in Fig. 1, Fig. 2, Fig. 3, Fig. 4.

The “success group” presented an ioPTH decrease variation from 26.89% to 92.40%, with an average result of 71.51%; on the other hand, the “failure group” presented a variation of 50.93%–85.60%, with an average result of 70.4%. To assess possible differences between groups, the Mann-Whitney test was applied, however, no significant differences could be detected (*p* = 0.990).

According to the absolute PTH value of the first PO, in the “success group” could be found a range from 4 pg/mL to 151.6 pg/mL, with an average result of 52.69 pg/mL, however, the “failure group” showed a variation from 17 pg/mL to 95.9 pq/mL, presenting an average result of 54.55 pg/mL. Once again, the values showed no statistical difference (*p* = 0.27).

## Discussion

In the present study, subtotal parathyroidectomy performed for patients with HPTRT proved to be effective in resolving hypercalcemia between 87% of patients.

The historical evolution of HPTRT surgical treatment has been evolved to minimize relapses and to avoid definitive hypoparathyroidism. Total parathyroidectomy has demonstrated high success for HPTTR, while subtotal parathyroidectomy has been minimizing its main complication, such as hypoparathyroidism. However, several studies failed to determine whether one technique is superior to the other.^13^, ^15^

Thus, seeing that there was no superiority among surgical techniques, the medical team must choose among them, nevertheless, individual characteristics of each patient (surgical risk and comorbidities) and intraoperative findings (size and position of the glands) must also be considered by the medical team.

Despite the fact that subtotal parathyroidectomies may have an increased risk of recurrent HPT (from 5% to 30%) compared to total parathyroidectomies, the subtotal parathyroidectomies are associated with lower rates of hypoparathyroidism and preservation of renal function.^14^, ^16^, ^17^ There is evidence that removal of parathyroid tissue accompanied by an abrupt decrease in PTH is related to worsening renal function, possibly due to the loss of the vasodilatory effect of PTH in afferent renal arteriole and vasoconstrictor effect in efferent renal arteriole.^15^, ^18^ Studies concerning the link between extension of the surgical procedure and renal function showed a worsening renal transplant function in the total parathyroidectomy group when data are compared to the subtotal parathyroidectomy group.^11^, ^16^

Currently, even if the sample size is limited to the statistical analysis, no patient had renal graft loss or hypoparathyroidism after six-months of follow-up, and the therapeutic failure rate showed 13% of effectiveness, and these data are in accordance to the average results presented in literature.

Recurrent prevalence of HPTRT can be treated with drug control of PTH and calcemia, but surgical re-approach may be required, and possible complications of cervical reoperation should be considered. In subtotal parathyroidectomy, the risks of neck re-approach are more pressing, whereas in total parathyroidectomy, graft region re-approach has lower morbidity. For this reason, intrinsic factors and intraoperative findings are important in the definition of surgical approach, since glands that are situated in complex topographies should be resected.

Particularly, regarding parathyroidectomy in patient with HPTRT, this procedure should be performed with caution in these patients. It is not unsual to observe glands of different pathological aspects, owing to the action of renal transplantation that decreases parathyroid hyperplasia. Some patients may have slightly altered macroscopic parathyroid glands, and within this circumstance, a subtotal parathyroidectomy should be performed. This step must be taken due to the 10.7-fold increased risk of permanent hypoparathyroidism in patients with small glands (<100 mm^3^) undergoing auto-transplantation in total parathyroidectomy.^14^ However, the surgeon should always be able to perform both options with a treatment model that may be individualized to each patient.

The use of ioPTH as an auxiliary method in HPTRT surgeries is not so relevance according to an analysis of this cohort, being dosage of intraoperative and dosage of first day after surgery unable to predict long-term success.

The literature presented studies that demonstrate the efficiency of ioPTH in parathyroidectomy of renal patients.^13^, ^15^ However, such studies used patients submitted to total and subtotal parathyroidectomy. In total parathyroidectomy, ioPTH demonstrated that all hyperplastic tissue has been resected and has the ability to evidence the presence of supernumerary glands, in other words, to prevent the persistence/recurrence of HPTRT. In patients undergoing subtotal parathyroidectomy, tissue preservation in the cervical region enables immediate functioning of the preserved tissue and precludes any analysis of PTH decay during surgery.

## Conclusion

Subtotal parathyroidectomy is an effective procedure for the treatment of HPTRT and safe in relation to the rates of hypoparathyroidism and of loss of renal transplantation. Monitoring of decrease in ioPTH and PTH values in the first postoperative period is unable to predict surgical success.

## Financial support

None.

## Declaration of competing interest

The authors declare no conflicts of interest.

## References

[1] Kandil E., Florman S., Alabbas H. (2010). Exploring the effect of parathyroidectomy for tertiary hyperparathyroidism after kidney transplantation. Am J Med Sci.

[2] Evenepoel P., Claes K., Kuypers D., Maes B., Bammens B., Vanrenterghem Y. (2004). Natural history of parathyroid function and calcium metabolism after kidney transplantation: a single-centre study. Nephrol Dial Transplant.

[3] Copley J.B., Wüthrich R.P. (2011). Therapeutic management of post-kidney transplant hyperparathyroidism. Clin Transplant.

[4] Dulfer R.R., Franssen G.J.H., Hesselink D.A., Hoorn E.J., van Eijck C.H.J., van Ginhoven T.M. (2017). Systematic review of surgical and medical treatment for tertiary hyperparathyroidism. Br J Surg.

[5] Tominaga Y., Kakuta T., Yasunaga C., Nakamura M., Kadokura Y., Tahara H. (2016). Evaluation of parathyroidectomy for secondary and tertiary hyperparathyroidism by the Parathyroid Surgeons’ Society of Japan. Ther Apher Dial.

[6] Goldsmith D., Covic A., Vervloet M. (2015). Should patients with CKD stage 5D and biochemical evidence of secondary hyperparathyroidism be prescribed calcimimetic therapy? An ERA-EDTA position statement. Nephrol Dial Transplant.

[7] Neves M.C.D., Rocha L.A.D., Cervantes O., Santos R.O. (2018). Initial surgical results of 500 Parathyroidectomies for Hyperparathyroidism related to chronic kidney disease - mineral and bone disorder. J Bras Nefrol.

[8] Meng C., Martins P., Frazão J., Pestana M. (2017). Parathyroidectomy in persistent post- transplantation hyperparathyroidism: single-center experience. Transplant Proc..

[9] Goldenstein P.T., Elias R.M., Carmo L.P.F. (2013). Parathyroidectomy improves survival in patients with severe hyperparathyroidism: a comparative study. PLoS One.

[10] Sampaio Ede A., Moysés R.M. (2011). Parathyroidectomy in CKD. J Bras Nefrol.

[11] Schlosser K., Endres N., Celik I., Fendrich V., Rothmund M., Fernández E.D. (2007). Surgical treatment of tertiary hyperparathyroidism: the choice of procedure matters!. World J Surg.

[12] Neves M.C., Ohe M.N., Rosano M. (2012). A 10-year experience in intraoperative parathyroid hormone measurements for primary hyperparathyroidism: a prospective study of 91 previous unexplored patients. J Osteoporos.

[13] Ohe M.N., Santos R.O., Kunii I.S. (2013). Intraoperative PTH cutoff definition to predict successful parathyroidectomy in secondary and tertiary hyperparathyroidism. Rev Bras Otorrinolaringol.

[14] Jäger M.D., Emmanouilidis N., Jackobs S. (2014). Presence of small parathyroid glands in renal transplant patients supports less-than-total parathyroidectomy to treat hypercalcemic hyperparathyroidism. Surgery.

[15] El-Husseini A., Wang K., Edon A. (2018). Value of intraoperative parathyroid hormone assay during parathyroidectomy in dialysis and renal transplant patients with secondary and tertiary hyperparathyroidism. Nephron.

[16] Jäger M.D., Kaaden S., Emmanouilidis N. (2011). Effect of incomplete parathyroidectomy preserving entire parathyroid glands on renal graft function. Arch Surg.

[17] Santos R.D., Rossi A., Coyne D., Maw T.T. (2019). Management of post-transplant hyperparathyroidism and bone disease. Drugs.

[18] antos J.H.Z., Enout M.J.R., Shimozono A.T. (2018). Evolution of renal function in patients with primary hyperparathyroidism submitted to parathyroidectomy. Arch Head Neck Surg.

